# Pre-Treatment Neutrophil Count as a Predictor of Antituberculosis Therapy Outcomes: A Multicenter Prospective Cohort Study

**DOI:** 10.3389/fimmu.2021.661934

**Published:** 2021-07-02

**Authors:** Anna Cristina C. Carvalho, Gustavo Amorim, Mayla G. M. Melo, Ana Karla A. Silveira, Pedro H. L. Vargas, Adriana S. R. Moreira, Michael S. Rocha, Alexandra B. Souza, María B. Arriaga, Mariana Araújo-Pereira, Marina C. Figueiredo, Betina Durovni, José R. Lapa-e-Silva, Solange Cavalcante, Valeria C. Rolla, Timothy R. Sterling, Marcelo Cordeiro-Santos, Bruno B. Andrade, Elisangela C. Silva, Afrânio L. Kritski, the RePORT Brazil consortium

**Affiliations:** ^1^ Laboratório de Inovações em Terapias, Ensino e Bioprodutos (LITEB), Instituto Oswaldo Cruz, Fundação Oswaldo Cruz, Rio de Janeiro, Brazil; ^2^ Programa Acadêmico de Tuberculose da Faculdade de Medicina, Universidade Federal do Rio de Janeiro, Rio de Janeiro, Brazil; ^3^ Department of Biostatistics, Vanderbilt University Medical Center, Nashville, TN, United States; ^4^ Laboratório de Micobacteriologia Molecular, Faculdade de Medicina e Complexo Hospitalar Hospital Universitário Clementino Fraga Filho—Instituto de Doenças do Tórax da Universidade Federal do Rio de Janeiro, Rio de Janeiro, Brazil; ^5^ Multinational Organization Network Sponsoring Translational and Epidemiological Research (MONSTER) Initiative, Salvador, Brazil; ^6^ Instituto Brasileiro para Investigação da Tuberculose, Fundação José Silveira, Salvador, Brazil; ^7^ Gerência de Micobacteriologia, Fundação de Medicina Tropical Doutor Heitor Vieira Dourado, Manaus, Brazil; ^8^ Laboratório de Inflamação e Biomarcadores, Instituto Gonçalo Moniz, Fundação Oswaldo Cruz, Salvador, Brazil; ^9^ Faculdade de Medicina, Universidade Federal da Bahia, Salvador, Brazil; ^10^ Division of Infectious Diseases, Department of Medicine, Vanderbilt University School of Medicine, Nashville, TN, United States; ^11^ Secretaria Municipal de Saúde do Rio de Janeiro, Rio de Janeiro, Brazil; ^12^ Instituto Nacional de Infectologia Evandro Chagas, Fundação Oswaldo Cruz, Rio de Janeiro, Brazil; ^13^ Curso de Medicina, Escola Bahiana de Medicina e Saúde Pública, Salvador, Brazil; ^14^ Curso de Medicina, Universidade Salvador (UNIFACS), Salvador, Brazil; ^15^ Laboratório Reconhecer Biologia, Centro de Biociência e Biotecniologia, Universidade Estadual do Norte Fluminense Darcy Ribeiro, Rio de Janeiro, Brazil

**Keywords:** tuberculosis, neutrophils, treatment outcome, biomarker, neutrophil count

## Abstract

**Background:**

Neutrophils have been associated with lung tissue damage in many diseases, including tuberculosis (TB). Whether neutrophil count can serve as a predictor of adverse treatment outcomes is unknown.

**Methods:**

We prospectively assessed 936 patients (172 HIV-seropositive) with culture-confirmed pulmonary TB, enrolled in a multicenter prospective cohort study from different regions in Brazil, from June 2015 to June 2019, and were followed up to two years. TB patients had a baseline visit before treatment (month 0) and visits at month 2 and 6 (or at the end of TB treatment). Smear microscopy, and culture for *Mycobacterium tuberculosis* (MTB) were performed at TB diagnosis and during follow-up. Complete blood counts were measured at baseline. Treatment outcome was defined as either unfavorable (death, treatment failure or TB recurrence) or favorable (cure or treatment completion). We performed multivariable logistic regression, with propensity score regression adjustment, to estimate the association between neutrophil count with MTB culture result at month 2 and unfavorable treatment outcome. We used a propensity score adjustment instead of a fully adjusted regression model due to the relatively low number of outcomes.

**Results:**

Among 682 patients who had MTB culture results at month 2, 40 (5.9%) had a positive result. After regression with propensity score adjustment, no significant association between baseline neutrophil count (10^3^/mm^3^) and positive MTB culture at month 2 was found among either HIV-seronegative (OR = 1.06, 95% CI = [0.95;1.19] or HIV-seropositive patients (OR = 0.77, 95% CI = [0.51; 1.20]). Of 691 TB patients followed up for at least 18 months and up to 24 months, 635 (91.9%) were either cured or completed treatment, and 56 (8.1%) had an unfavorable treatment outcome. A multivariable regression with propensity score adjustment found an association between higher neutrophil count (10^3^/mm^3^) at baseline and unfavorable outcome among HIV-seronegative patients [OR= 1.17 (95% CI= [1.06;1.30]). In addition, adjusted Cox regression found that higher baseline neutrophil count (10^3^/mm^3^) was associated with unfavorable treatment outcomes overall and among HIV-seronegative patients (HR= 1.16 (95% CI = [1.05;1.27]).

**Conclusion:**

Increased neutrophil count prior to anti-TB treatment initiation was associated with unfavorable treatment outcomes, particularly among HIV-seronegative patients. Further prospective studies evaluating neutrophil count in response to drug treatment and association with TB treatment outcomes are warranted.

## Introduction

Neutrophils have been implicated in TB pathogenesis (1, 2). Several studies in animal models as well as in humans have revealed a prominent role of neutrophils in tissue damage during active TB, leading to more severe clinical presentations (3). There are increasing evidence that the neutrophil number and degree of neutrophil activation directly correlate with the degree of lung destruction seen in pulmonary TB (4). More recently, Ndlovo et al. described that high peripheral neutrophil count and low CD15 expression directly correlated with more severe lung damage on chest x-ray (5).

Neutrophils are also relevant for TB diagnosis, as several transcriptomic signatures indicate enriched pathways involving this cell type that can be used to distinguish active from latent TB infection (6).

Although several studies have evaluated the association between blood neutrophils and unfavorable TB treatment outcome (4, 7–14), there are limited data from well-powered prospective studies. A key advantage of neutrophils as a biomarker is that they can be measured in clinical laboratories in resource-limited settings.

To evaluate the relationship between blood neutrophil count and poor TB treatment outcome (i.e., treatment failure, mortality or relapse), we analyzed patients with positive *Mycobacterium tuberculosis* (MTB) culture of respiratory samples enrolled in the Regional Prospective Observational Research on Tuberculosis (RePORT) - Brazil cohort (15).

## Methods

### Study Design

We performed a multicenter prospective observational cohort study of individuals with culture-confirmed pulmonary TB. All study participants were enrolled in Regional Prospective Observational Research on Tuberculosis (RePORT)-Brazil (15), between June 2015 and June 2019, and were followed for up to two years. RePORT-Brazil includes two prospective cohorts: patients with pulmonary TB, and their close contacts. Objectives include the identification of clinical, radiological and laboratory variables associated with TB treatment outcome, and predictive of TB disease among contacts. Study sites were located in Rio de Janeiro State, Southeastern region (Centro Municipal de Saúde de Duque de Caxias - site A; Instituto Nacional de Infectologia Evandro Chagas - site D; Clínica da Familia Rinaldo Delamare-site E); in the cities of Manaus, Northern region (site B), and Salvador, Northeastern region (site C).

These sites represent Brazilian cities with the highest TB burden (16). Site B and site D are HIV reference centers. In this study, we enrolled participants who had microbiologically confirmed TB, were over 18 years of age and provided written informed consent. Those who received anti-TB drugs (including fluoroquinolones) for more than 7 days in the 30 days prior to TB diagnosis were excluded. A trained nursing team conducted patient interviews and collected sociodemographic and clinical data. Participants underwent the following tests: chest radiograph, HIV testing, CD4 and viral load (if HIV-seropositive), complete blood count, glycated hemoglobin (HbA1c), sputum smear microscopy, Xpert-MTB-RIF (if available), mycobacterial culture (Lowenstein-Jensen medium or BD BACTEC MGIT) and drug susceptibility testing (proportion method or BD BACTEC GIT). The study participants had a baseline visit (M0) and follow-up visits at month 2 (M2) and month 6 (M6 or at the end of TB treatment), when clinical status was reassessed, and new smear/culture tests were performed. Only drug susceptible TB patients followed up at least 2 months were included in the microbiological analysis; participants received standard 6-month treatment for TB, consisting of isoniazid, rifampicin, pyrazinamide, and ethambutol for 2 months followed by isoniazid and rifampicin for at least 4 months, following Brazilian National guidelines for TB control (16).

### Study Definitions and Procedures

Complete blood counts were performed only at baseline. Anemia was defined as hemoglobin levels <12 g/dL for females and <13.5 g/dL for males. Diabetes mellitus (DM) was diagnosed according to the baseline HbA1c, following the American Diabetes Association (ADA) guidelines (17). Patients with HbA1c ≥ 5.7% were classified as having dysglycemia and, among those, they were classified as having DM if HbA1c ≥ 6.5%, prediabetes (pre-DM) if HbA1c was between 5.7% and 6.4%. Patients with HbA1c lower than 5.7% were considered normoglycemic. Data on other variables such as age, sex, HIV serology, race/skin color (self-reported), body mass index (BMI), BCG scar, education level, income status, tobacco smoking status, alcohol consumption (according to CAGE questionnaire), illicit drug use, cavitation on chest radiograph and study site were obtained from all participants. Information about the symptoms of TB was also obtained at the baseline and at M2 and M6 visits. Neutrophil count at baseline as well as sputum smear and MTB culture results at month 2 were recorded. Treatment outcome was defined as either unfavorable (death from any cause, treatment failure or TB recurrence) or favorable (cure or treatment completion) following the World Health Organization (WHO) guidelines (18). Patients lost to follow-up were not included in the analysis of neutrophil count and TB treatment outcome.

### Data Analysis

Quantitative variables were presented as medians and interquartile ranges (IQR) and qualitative variables as percentages. The effect of baseline characteristics on the outcome of interest were computed *via* univariable logistic regression. P-values were computed *via* Wald tests.

Logistic regression analysis was also used to estimate associations between neutrophil count at baseline with smear microscopy, culture conversion at month 2 and TB treatment outcome, adjusting for HIV serology (and an interaction term). This was denoted as unadjusted logistic regression, since it did not take into account potential confounders. Data on the following clinical factors were collected: COPD; renal disease; hypertension; chemotherapy or radiotherapy; immunosuppressor drug (corticosteroid) and were included in the propensity score model.

Because the number of outcomes was relatively low, we were unable to fit a fully adjusted regression model including all covariates of interest. Instead, we fit a logistic regression model using propensity score adjustment (19). Propensity score adjustment, which may be seen as a variable reduction technique, is a two-step procedure: 1) in the first step, the propensity score is estimated by regressing the exposure variable on a set of covariates and 2) in the second step, the outcome of interest is regressed on the exposure while controlling for the estimated propensity score, obtained in step 1. By this way any extra confounder is included in the outcome model *via* the estimated propensity score.

For our setting, the propensity score was fit *via* an ordinal regression, by regressing the exposure variable on a set of pre-specified covariates. We followed simulation results from (20), which showed better performance (i.e., smaller errors) when a fine stratification (20 strata) of the exposure was used and modelled *via* robust approaches, such as ordinal regression, that required no assumptions about underlying distributions. This propensity score model is constructed in the step 1 outlined above. It used the following covariates for adjustment: sex, age, race, smoking status, alcohol consumption, education level, income status, HIV serology status, cavitation on chest radiograph, DOT use (for TB treatment outcome only), study site, and (log-transformed) platelets, lymphocytes, glycated hemoglobin and hemoglobin values. All variables were selected a priori, based on data from the literature and clinical plausibility. Restricted cubic splines with 3 knots equally spaced were used to relax the linearity assumption, and an interaction between age and sex was also added to the propensity score model.

This two-step procedure allowed us to fit a larger set of covariates in the first step (to estimate the propensity score) and a smaller model in step 2 (21). Our main model regressed the outcome of interest on neutrophil count at baseline, HIV status, and on the estimated propensity score. We also added an interaction term of neutrophil count and HIV status. This was denoted as an adjusted model, since it took into account several potential confounders in the estimated propensity score. We did not stratify the exposure in this second and final step; neutrophil count was in its natural, continuous scale. To allow for non-linearity, the estimated propensity score was in the logit scale.

Finally, results were expressed in terms of point estimates and 95% confidence intervals; odds ratios were calculated, for interpretation purposes, for every 1,000 change of neutrophil count. Missing values were imputed 20 times *via* Markov Carlo Chained Equations (22) and final estimates were obtained *via* Rubin’s rule (23). All analyses were performed using the statistical software R (24).

### Ethical Approval

The protocol, consent form, and study documents were approved by the institutional review boards at the study sites. Participation was voluntary, and written informed consent was obtained from all participants.

## Results

### Study Population

Sample size for all analysis are displayed in **Figure S1**. Population demographics and laboratory values are depicted in **Table 1**. A total of 936 patients were included in the analysis, of whom 172 (18.4%) were HIV-seropositive. The overall median age at enrollment was of 35 years (IQR = 25.0; 49.0) and most were non-black (74.0%), male (66.1%), had anemia (57.5%), and a BCG scar (86.5%); 45.3% were alcohol users, and 23.1% had DM. Compared to HIV-seronegative patients, as depicted in **Tables S1** and **S2**, HIV-seropositive participants were more likely to be non-black (OR = 2.22; 95% CI = 1.44; 3.54), male (OR = 1.94; 95% CI = 1.33;2.89), more likely to be treated in HIV reference centers, sites (B and D) (OR= 22.7; 95% CI = 11.0;55.4 and OR= 7.33; 95% CI = 3.25;19.0, respectively) and to have anemia (OR = 2.57; 95% CI= 1.78;3.78). On the other hand, HIV-seropositive individuals with TB were less likely to be tobacco smokers (OR= 0.61; 95% CI = 0.39;0.93); alcohol users (OR= 0.52; 95% CI = 0.36;0.74); BCG vaccinated (OR = 0.48; 95% CI = 0.32;0.74); have lung cavitary lesions on chest X-ray (OR = 0.20; 95% CI = 0.13;0.29), have high lymphocytes count (OR = 0.91; 95% CI= 0.88;0.94) and high glycosylated hemoglobin (OR= 0.79; 95% CI= 0.70;0.89).

**Table 1:** Sociodemographic characteristics and laboratory values for the study population.

	[ALL]
	*N=936*
**Sociodemographic characteristics**	
Age at enrollment:	35.0 [25.0;49.0]
Race/skin color: Non-black	692 (74.0%)
Sex: Male	619 (66.1%)
Smoking: Yes	213 (22.8%)
Smoking (years)	15.0 [5.0;25.0]
Alcohol: Yes	424 (45.3%)
HIV: Positive	172 (18.4%)
BCG scar: Yes	809 (86.5%)
Alcohol (years)	13.0 [6.00;27.0]
X-ray cavitation: Yes	465 (50.0%)
Literate: Yes	889 (95.1%)
Education (years):	9.00 [6.00;12.0]
Income: more than minimum wage	299 (32.6%)
HIV treatment^a^: Yes	127 (73.8%)
CD4 (cells/mm^3^)	135 [63.5;298]
Study site:	
A	179 (19.1%)
B	267 (28.5%)
C	240 (25.6%)
D	124 (13.2%)
E	126 (13.5%)
**Laboratory values**	
Leukocytes (10^3^/mm^3^)	8.49 [6.53;10.7]
Neutrophils (10^3^/mm^3^)	6.05 [4.39;7.97]
Lymphocytes (10^2^/mm^3^)	15.5 [11.8;19.5]
Hemoglobin (g/dL)	12.1 [10.7;13.4]
Anemia: Yes	534 (57.5%)
Platelet (10^4^/mm^3^)	38.5 [30.9;47.9]
Glycosylated Hemoglobin (%) (n= 927)	5.80 [5.40;6.40]
Glycosylated Hemoglobin (%):	
<5.7	416 (44.9%)
5.7-6.5	297 (32.0%)
≥6.5	214 (23.1%)

Values are represented as frequency (%) or median with interquartile range (IQR). Smoking: current smoker (Yes/No); Alcohol: current (Yes/No); HIV (Positive/Negative); Literate: literacy (Yes/No); Income: monthly salary; CD4: CD4 count at baseline; Study site: sites covered by RePORT. Anemia: hemoglobin levels <12 g/dL for female and <13.5 g/dL for male; ^a^ART frequency was calculated among the persons living with HIV; Study sites: A – Caxias Health Center/Rio de Janeiro, B- Tropical Medicine Foundation/Manaus; C: Jose Silveira Foundation/Salvador; D: Evandro Chagas Institute-Rio de Janeiro; E: Rocinha – Municipality of Rio de Janeiro.

The median neutrophil count at baseline was 6,050 cells/µL (IQR = 4,390;7,970). HIV-seropositive patients had lower neutrophil count, 5,050 cells/µL (IQR = 3,590;7,250) compared to HIV-seronegative, 6,120 cells/µL (IQR = 4,630;8,090). This difference was statistically significant at the 5% level (OR=0.92; 95% CI = 0.86;0.97).

### Association Between Neutrophils and TB Bacillary Load in Sputum at Baseline

**Tables S3** and **S4** show univariable comparisons for association between sociodemographic and laboratory values with smear positivity at study baseline, respectively. Current tobacco smokers, alcohol users, and those with lung cavitation had higher odds of being smear positive at M0 (OR= 1.62; 95% CI= 1.07, 2.52; OR=1.52; 95% CI=1.09-2.13; and OR = 3.10; 95% CI = 2.18, 4.45, respectively). Leukocytes (OR = 1.11 [1.05;1.17]), neutrophil (OR=1.11 [1.04;1.18]), and platelet (OR=1.02 [1.01;1.03]) counts were also associated with positive smear results. HIV-seropositive patients were less likely to be smear positive at baseline (OR = 0.34, 95% CI = 0.23;0.49).

Unadjusted and adjusted logistic regression models were used to estimate the association between neutrophil count at baseline with the odds of being smear positive at baseline. Results suggested highly different neutrophil counts between seropositive and seronegative patients (p-value < 0.001). The adjusted regression showed a strong association between neutrophil count and smear result at baseline among HIV-seronegative patients: OR = 1.16, 95% CI = 1.07, 1.26. This association was not seen among HIV-seropositive patients: OR = 0.99, 95% CI = 0.82, 1.20, as depicted in **Table 2** and **Figure S2**.

**Table 2:** Association between neutrophil count at baseline and the outcome of interest.

Outcome variable	Strata (size)	Unadjusted analysis	Adjusted analysis^†^
Positive Smear result at baseline	HIV-seronegative (756)	1.16 [1.07;1.25]	1.16 [1.07; 1.26]
	HIV-seropositive (171)	0.99 [0.91;1.08]	0.99 [0.82; 1.20]
Positive Smear result at month 2	HIV-seronegative (564)	1.08 [1.01; 1.16]	1.08 [1.00; 1.17]
	HIV-seropositive (145)	0.78 [0.65; 0.94]	0.78 [0.62; 0.99]
Positive MTB culture at month 2	HIV-seronegative (540)	1.08 [0.98; 1.20]	1.06 [0.95; 1.19]
	HIV-seropositive (131)	0.78 [0.53; 1.15]	0.77 [0.51; 1.20]
Unfavorable TB treatment outcome	HIV-seronegative (577)	1.19 [1.08; 1.32]	1.17 [1.06; 1.30]
	HIV-seropositive (144)	0.99 [0.87; 1.13]	0.98 [0.77; 1.24]

Both analysis control for HIV serology and its interaction with baseline neutrophil. ^†^ Model adjusting for the propensity score, which was regressed on the following covariates: sex, age, race, smoking status, alcohol consumption, education level, income status, cavitation on chest radiograph, study site, HIV status, DOT (for unfavorable TB treatment outcome) and (log-transformed) platelets, lymphocytes, glycated hemoglobin and hemoglobin values; results expressed in odds ratios (OR) with 95% confidence intervals.

### Factors Associated With No Sputum Smear Conversion During TB Treatment

A total of 713 patients had sputum smear result at M2, from which 126 (17.7%) were still smear positive. Univariable logistic regressions are presented in **Tables S5** and **S6**, for sociodemographic and laboratory values, respectively. Increased age (OR = 1.03, 95% CI = 1.01;1.04), years of smoking (OR=1.04 95% CI= 1.01;1.07); alcohol use (OR= 1.03, 95% CI= 1.01;1.05); anemia (OR=1.67, 95% CI =1.11;2.53), and DM (OR=1.81, 95% CI =1.10;2.96) had higher chance of having smear positive sputum at M2. HIV-seropositive patients were also on average more likely to be smear positive at M2 (OR = 1.74, 95% CI = 1.11;2.68). Univariable analysis showed that neutrophil count was not statistically associated with higher odds of being sputum smear positive at month 2, as suggested by the boxplots in **Figure S3** (OR = 1.00, 95% CI = 0.94; 1.07) and depicted in **Table S6**.

Neutrophil count, however, differed significantly by HIV status (p-value < 0.01). Smear positive sputum at M2 was associated with higher neutrophil count among HIV-seronegative patients in both unadjusted and adjusted (by the propensity score) analysis (OR = 1.08, 95% CI = 1.01;1.16 and OR = 1.08, 95% CI = 1.00;1.17, respectively). Interestingly, among HIV-seropositive patients, the unadjusted analysis suggested an association between higher neutrophil count and lower odds of a smear positive sputum at M2 (OR = 0.78, 95% CI = 0.65; 0.94). The adjusted analysis showed that for every increase in the neutrophil count by 1,000 units, the odds of being sputum smear positive at month 2 decreased on average by ~22% (OR = 0.78, 95% CI = 0.62;0.99). Results for both unadjusted and adjusted regression with propensity score adjustment are displayed in **Table 2**.

It is important to note that the relationship between neutrophil and smear positive at month 2 may not be linear, in the sense that high and low neutrophil counts would lead to poor prognosis (especially among HIV-seronegative patients). To explore this further, we re-fitted the model, using restricted cubic splines, with 3 knots equally spaced, to relax the linearity assumption. **Figure S4** provides two plots of the log odds of having a positive smear result at month 2 by neutrophil count, for both HIV-seropositive and HIV-seronegative patients. The figure shows that, for HIV-seropositive patients, higher neutrophil count is associated with better prognosis (smaller odds of being smear positive at month 2). This increment, however, may not be linear; there is large uncertainty when neutrophil count increase (wider 95% confidence interval - grey area). Among HIV-seronegative patients, we see the opposite trend: higher neutrophil count lead to worse prognosis. The trend, again, may not be linear, as data become less frequent for higher values, increasing uncertainty (again, wider 95% confidence intervals as neutrophil count increases).

In summary, both plots support our results: HIV-seropositive patients with higher neutrophil count seem less likely to be smear positive at month 2, while HIV-seronegative patients with higher neutrophil count seem more likely to be smear positive at month 2. Although the relationship may not be linear (more data are needed to address this issue), it seems unlikely that low neutrophil count lead to poor prognosis among HIV-seronegative patients.

### Characteristics Associated With No Sputum Culture Conversion During TB Treatment

A total of 886 patients had a visit reported at M2. Of these, 176 did not provide a respiratory sample, 631 had negative culture results for MTB, 28 patients had contaminated culture and 51 had positive results, from which 40 were found to be positive for MTB and 11 for nontuberculous mycobacterial (NTM). A total of 682 TB patients with reported positive or negative culture results were included in the analysis, of which 40 (7 HIV-seropositive) were positive for MTB at M2. The 11 NTM cultures were not included as positive.

Univariable logistic regressions are presented in **Tables S7** and **S8**, for demographic and laboratory values, respectively, stratified by MTB status. The following variables were associated with positive MTB culture at M2: older age (OR= 1.05; 95% CI= 1.05; 1.07) and years of smoking (OR=1.04; 95% CI= 1.01; 1.07). HIV serology status was not associated with culture result at M2 (OR = 0.83, 95% CI = 0.33;1.83, for HIV-seropositive as reference level).

Unadjusted and propensity score adjusted logistic regressions did not show evidence of association between neutrophil count and culture conversion at M2, as indicated by the boxplots in **Figure S5** (e.g., OR=1.06, 95% CI= 0.95;1.19 and OR=0.77, 95% CI= 0.51;1.20, for seronegative and seropositive patients, respectively, in the adjusted analysis). Results for both unadjusted and adjusted regressions are displayed in **Table 2**.

### Blood Neutrophil Count as a Predictor of Unfavorable Anti-TB Treatment Outcomes

A total of 691 TB patients, followed-up for at least 18 months and maximum of 24 months, were used for analysis. Among them, 635 (91.9%) were either cured or completed treatment without bacteriologic confirmation of cure, while 56 (8.1%) developed an unfavorable treatment outcome: 22 died (4 related to TB, 13 not caused by TB and 5 with reasons that are not clearly related to TB), 25 had treatment failure and 9 TB recurrence.

**Figure 1:**
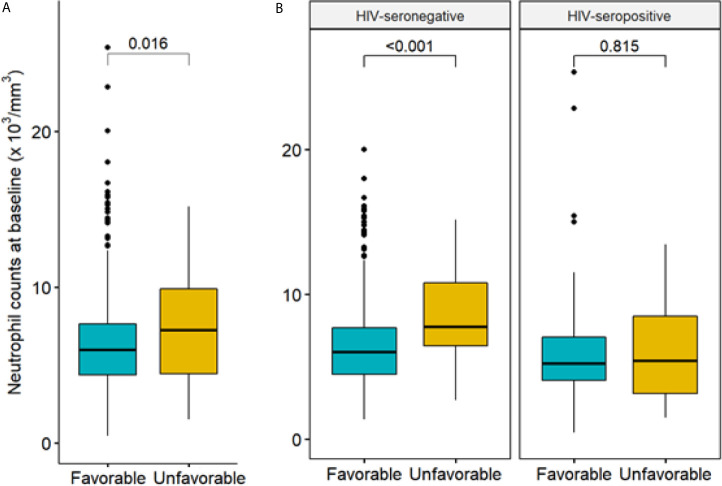
Neutrophil count at baseline by treatment outcome. **(A)** Comparison of neutrophil count at baseline by treatment outcome (favorable/unfavorable, among N=691 patients); **(B)** comparisons of neutrophil count by treatment outcome, stratified by HIV status (577 seronegative patients, with 34 unfavorable outcome, and 114 seropositive patients, with 22 unfavorable outcome). Favorable treatment: cure or treatment completion. Unfavorable treatment: death, failure, recurrence. P-value computed via Wald test.

Their baseline sociodemographic and laboratory values are displayed in **Tables 3** and **4**, stratified by TB treatment outcome. The following variables were associated with an unfavorable TB outcome: HIV infection (OR= 3.82, 95% CI= 2.11;6.80), treated at a HIV reference center (site B) (OR= 3.82, 95% CI= 1.64;10.6), anemia (OR= 1.78, 95% CI= 1.00;3.30), DM (OR= 3.30, 95% CI= 1.65;6.83), pre-DM (OR=2.11, 95% CI=1.04;4.39). Patients with higher neutrophil count were also more likely to have unsuccessful treatment in a univariable analysis (OR= 1.10, 95% CI= 1.02; 1.19); i.e., increasing neutrophil count by 1,000 units, the odds of developing an unfavorable TB treatment increased, on average, by 10%, as suggested by **Figure 1**.

**Table 3:** Sociodemographic characteristics, stratified by TB treatment outcome.

	Favorable treatment outcome	Unfavorable treatment outcome	P-value
	*N=635*	*N=56*	
Age at enrollment:	37.0 [26.0;49.0]	36.5 [25.0;49.5]	0.964
Race:/skin color: Non-black	471 (74.3%)	42 (75.0%)	0.925
Sex: Male	404 (63.6%)	37 (66.1%)	0.725
HIV: Yes	92 (14.5%)	22 (39.3%)	<0.001
HIV treatment: Yes	72 (88.9%)	15 (93.8%)	0.631
CD4 (cells/mm^3^)	150 [63.5;358]	112 [62.8;161]	0.059
Smoking: Yes	131 (20.6%)	11 (19.6%)	0.884
Smoking (years)	16.0 [6.00;26.0]	11.0 [5.25;20.0]	0.173
Alcohol: Yes	281 (44.3%)	21 (37.5%)	0.334
BCG scar: Yes	549 (86.6%)	48 (85.7%)	0.827
Alcohol (years)	14.0 [7.00;28.0]	13.0 [5.50;23.0]	0.681
X-ray cavitation: Yes	328 (54.5%)	25 (48.1%)	0.378
Literate: Yes	32 (5.04%)	2 (3.57%)	0.687
Education (years):	10.0 [6.00;12.0]	9.00 [5.00;12.0]	0.265
Income: more than minimum wage	223 (35.9%)	18 (32.1%)	0.583
DOT: Yes	408 (64.7%)	34 (60.7%)	0.554
Study site:			
A	125 (19.7%)	6 (10.7%)	Ref.
B	154 (24.3%)	29 (51.8%)	0.001
C	163 (25.7%)	13 (23.2%)	0.327
D	85 (13.4%)	6 (10.7%)	0.528
E	108 (17.0%)	2 (3.57%)	0.261

Values are represented as frequency (%) or median with interquartile range (IQR). 95% confidence intervals are displayed. P-values computed via Wald tests. Smoking: current smoker (Yes/No); Alcohol: current (Yes/No) Literate: literacy (Yes/No); Income: monthly salary. CD4: CD4 count at baseline. Study site: sites covered by RePORT. Favorable outcome: cured or treatment completion; Unfavorable outcome: death, treatment failure, recurrence. OR, odds ratio; DOT, direct observed treatment. Study sites: A – Caxias Health Center/Rio de Janeiro, B- Tropical Medicine Foundation/Manaus; C: Jose Silveira Foundation/Salvador; D: Evandro Chagas Institute-Rio de Janeiro; E: Rocinha – Municipality of Rio de Janeiro.

**Table 4:** Laboratory values, stratified by TB treatment outcome.

	Favorable treatment outcome	Unfavorable treatment outcome	P-value
	*N=635*	*N=56*	
Neutrophils (10^3^/mm^3^)	5.98 [4.36;7.64]	7.24 [4.41;9.89]	0.014
Glycosylated Hemoglobin (%)	5.80 [5.40;6.30]	6.15 [5.75;6.73]	0.448
Platelet (10^4^/mm^3^)	38.7 [31.1;47.2]	39.6 [32.0;50.6]	0.176
Lymphocytes (10^2^/mm^3^)	15.7 [12.1;19.5]	14.4 [9.77;21.1]	0.870
Leukocytes (10^3^/mm^3^)	8.48 [6.49;10.4]	10.1 [6.45;13.2]	0.051
Hemoglobin (g/dL)	12.2 [11.1;13.4]	10.7 [9.50;12.6]	<0.001
Anemia: Yes	355 (56.2%)	39 (69.6%)	0.050
Glycosylated Hemoglobin (%):			
<5.7	294 (46.6%)	14 (25.0%)	Ref.
5.7-6.5	198 (31.4%)	20 (35.7%)	0.038
≥6.5	139 (22.0%)	22 (39.3%)	0.001

Values are represented as frequency (%) or median with interquartile range (IQR). 95% confidence intervals are displayed. P-values computed via Wald tests. Favorable outcome: cured or treatment completion; Unfavorable outcome: death, treatment failure, recurrence. Anemia: hemoglobin levels <12 g/dL for female and <13.5 g/dL for male. OR, odds ratio.

Neutrophil count was again statistically different between patients in opposite HIV serology groups (p-value < 0.01). Unadjusted regression analysis demonstrated a strong association between higher neutrophil count and unfavorable treatment outcomes (OR= 1.19, 95% CI= 1.08; 1.32), which was again detected in the logistic regression with propensity score adjustment (OR= 1.17, 95% CI= 1.06; 1.30). Among HIV-seropositive patients, however, which accounted for 61 subjects (22 with unfavorable outcome), no association between neutrophil count at M0 and unfavorable treatment outcome was observed in the unadjusted nor in the adjusted analysis (OR = 0.99, 95% CI = 0.87; 1.13 and OR = 0.98, 95% CI = 0.77; 1.24, respectively). Results for both unadjusted and adjusted regressions are displayed in **Table 2**.

As the unfavorable outcome group contained a very heterogenous group, we re-ran the analysis above for 4 additional settings: 1) discarding deaths that were not related to TB; 2) restricting the follow-up time to 9 months (so all 9 patients with TB recurrence were not included as an unfavorable TB treatment outcome); 3) re-analyzing unfavorable TB treatment outcome, under a mixed-effect perspective, with study site as a random effect in the main outcome regression model; and 4) re-analyzing unfavorable TB treatment outcome, adjusting for leukocytes. In this last analysis, (log-transformed) leukocytes were included in the propensity score model. Results for all of these analyses were similar to those provided above, with a statistically significant association between baseline neutrophil count with unfavorable treatment outcome among HIV-seronegative patients. These additional results are presented in **Table S9**.

**Figure 2:**
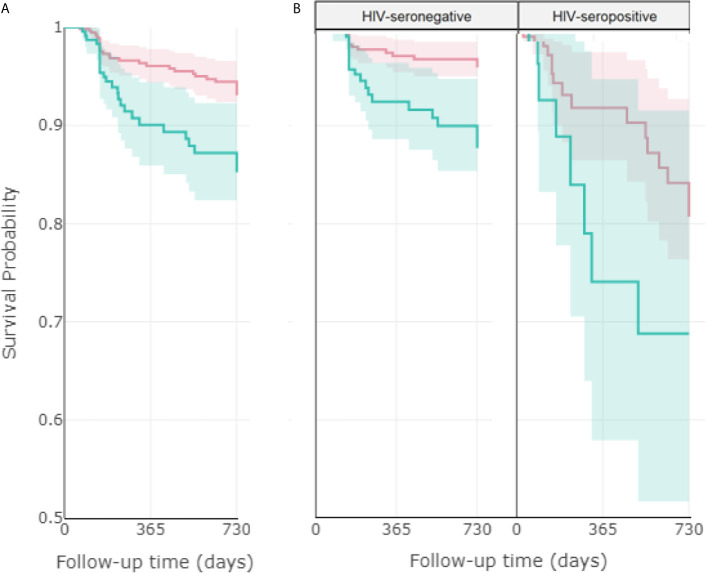
Association between neutrophil count at baseline with time until unfavorable treatment outcome. Kaplan-Meier curves comparing the impact of higher (>7500/mm^3^) and lower (<7500/mm^3^) neutrophil count at baseline on the probability of facing a favorable outcome, considering **(A)** the total population (919 patients), **(B)** stratified by HIV status (749 seronegative and 170 seropositive patients). The red line corresponds to low neutrophil count and the blue line corresponds to high neutrophil count, with their respective 95% confidence intervals. Favorable treatment: cure or treatment completion. Unfavorable treatment: death, failure, recurrence. Log rank p-values: 0.002 **(A)**, <0.001 (**B**, left), 0.1 (**B**, right).

**Figure 2** shows the Kaplan-Meier survival curve for time to unfavorable treatment outcome, by neutrophil count at baseline. The results indicated that higher neutrophil count at baseline was associated with higher chances of unfavorable treatment outcome. A Cox regression model with propensity score adjustment showed that neutrophil count differs substantially across HIV serology groups (p-value < 0.01). While the hazard of further developing an unfavorable treatment outcome was not significatively associated with neutrophil among HIV-seropositive patients, it was strongly associated with higher neutrophil count among HIV-seronegative patients. For every 1,000 units increase in neutrophil count at baseline, the hazard of further developing an unfavorable treatment outcome increased, on average, by 16% (HR= 1.16, 95% CI= 1.05; 1.27).

## Discussion

Neutrophils have received prominence in the pathogenicity of TB, although few prospective well-powered studies have analyzed the role of blood neutrophil count in predicting treatment outcome of pulmonary TB patients.

The present study, performed in a well-characterized cohort of culture-confirmed pulmonary TB patients, found that pre-treatment neutrophil count may serve as a reliable predictor of unfavorable TB treatment outcomes.

The association of neutrophil count with positive smear and cavity on chest radiograph was similar to those described by other groups (2, 5, 6). The association between higher neutrophil count and parenchymal findings in the chest radiogram was described by Kerkoff et al. (7) in HIV-seropositive and by de Mello et al. (2) in HIV-seronegative patients. Only in the study of Nodvlu et al. (5), who used radiological scores to analyze the extent of lung injury, the association between neutrophil count and chest radiographic findings was observed in both HIV-seropositive and negative patients.

In 2003, in addition to the presence of cavitation on chest radiograph, WHO guidelines recommended the sputum-smear examination at the end of the second month of treatment in patients with recently diagnosed pulmonary TB, and, if positive, the intensive phase of TB treatment should be extended (25). In recent years, it has been emphasized that culture conversion during treatment for TB has only a limited role in decision-making for advancing regimens into phase III trials or in predicting the outcome of treatment for individual patients (26, 27).

In our study, in univariable analysis, positive smear in month 2 was more frequent among older people, in patients infected with HIV, with anemia and DM. Neutrophil count was a statistically significant predictor at 5% level of positive smear in M2 only in TB/HIV-seronegative patients. Those results were also described by other authors (28–31).

In addition, in the univariable analysis we identified the following variables associated with positive smear or culture results at M2: older age, smoking and alcohol use. Those results were also described in other series (28, 32, 33). Caetano Mota et al. (28), in a retrospective cohort of 136 adult patients with pulmonary TB confirmed by positive culture for MTB on sputum, found that older age was independently associated with delayed smear conversion. Nijenbandring de Boer et al. (32) evaluated 89 active pulmonary TB patients with positive sputum culture. After adjustment for cavities on the chest radiograph and alcohol use, they found that current tobacco smoking compared to current non-smoking remained significantly associated with culture non-conversion at 60 days of treatment anti-TB. Volkmann T et al. (33), using data reported to the National Tuberculosis Surveillance System in USA on 207,307 adult TB cases, confirmed that excess alcohol use was associated with lower rates of sputum culture conversion.

In our study, there was no significant association between neutrophil count at the beginning of TB treatment and positive culture at month 2, irrespective of HIV status. De Melo et al. (2) and Brambhat et al. (12) described similar results in TB/HIV-seronegative patients. Nodvlu et al. (5) reported an association between positive culture in month 2 and the level of CD15 expression, but not with neutrophil count at the time of TB diagnosis, in both TB/HIV-seropositive and TB/HIV-seronegative patients.

In our large cohort of patients with pulmonary TB from high-burden cities in Brazil, the association of neutrophilia with unfavorable TB treatment outcome was confirmed in TB/HIV-seronegative patients, but not in TB/HIV-seropositive ones. Similar findings were described by other authors (5, 8, 13, 14). Barnes et al. (13), in USA, evaluating 191 consecutive HIV-seronegative adults with pulmonary TB found an association between neutrophil count and death. In the study of Lowe et al. (8), analyzing 855 TB patients with neutrophil count at baseline, neutrophilia was an independent risk for case fatality. Han et al. (14) carried out a retrospective study with 96 TB patients in South Korea. They found that high neutrophil/lymphocyte rate was also an independent predictor of in-hospital mortality.

On the other hand, HIV infection frequently reduces the neutrophil count (34). In addition, there is an impaired ability to phagocytize TB bacilli observed in TB/HIV-seropositive patients, but the phagocytosis capacity is restored after the use of antiretroviral therapy (35). In our cohort, median neutrophil count was lower in TB-HIV co-infected patients than in HIV-seronegative ones. Interestingly, we observed an inverse association between neutrophil count and smear-positivity in the sputum at M2 among HIV-seropositive patients, i.e., increasing neutrophil count was associated with a lower chance of smear-positivity. This finding may indicate the importance of neutrophilic activity in the response to MTB infection. However, above a certain threshold, not yet determined, this exacerbated neutrophilic response cause tissue damage and may compromise TB outcome.

In the analysis of the association between neutrophilia and unfavorable TB treatment outcomes, abandonment and/or loss of follow-up were not included, as they could be confounding variables (35). As described in systematic meta-analysis/reviews (36–38) and more recently by Demitto et al. (30), we observed an association between unfavorable outcome and HIV infection, diabetes mellitus and anemia. The association between high neutrophil count, anemia and diabetes mellitus with unfavorable treatment outcome may result from a decrease in the ability of neutrophils to kill mycobacteria followed by a nonspecific inflammatory cascade, characterized by the production of cytokines and/or exacerbated necrotic cell death (2, 30, 31). Such events produce the accumulation of neutrophils due to persistence of systemic inflammation; similar results were described recently with Covid-19 (39). In addition, recent transcriptomic data obtained in whole blood from TB patients confirmed the signatures of neutrophils correlated with the radiographic extent of TB disease that decreased during the first 2 months of TB treatment (5). Together with other simple diagnostic tests, neutrophil monitoring could be valuable as a rule-out test and for identifying patients at baseline with a higher chance of unfavorable treatment outcome.

### Limitations

Collinearity, if there were any, was not an issue while estimating the propensity score. This is because the objective at this stage is to predict the propensity score, not inference.

We did not analyze other non-specific biomarkers of inflammatory response associated with severe pulmonary TB, such as C-reactive protein, albumin and globulin (2, 8, 40, 41). These proteins, produced by the liver, act as homeostasis breakers and thus, could potentially be used as biomarkers, in addition to the blood neutrophil count. Anemia and HbA1c could be additional variables that could be assessed in future studies. Furthermore, we used only biomarkers obtained from blood analysis, and therefore, it was not possible to analyze specific biomarkers associated with an immune response in the lung. In addition, the extent of lung involvement was not evaluated (42).

### Conclusion

In our cohort, high blood neutrophil count at baseline was associated with unfavorable TB treatment outcome in HIV-seronegative TB patients, but not in HIV-seropositive patients. We found no association between baseline neutrophil count and positive MTB culture at month 2. The knowledge of the association between high neutrophil count and unfavorable treatment outcome can potentially help the clinician guide care to improve outcomes in these high-risk patients. Our results reinforce the need to carry out further prospective studies to analyze the impact of host directed therapy, such as anti-inflammatory drugs, in patients with abnormal levels of simple biomarkers, such as neutrophil count.

## Data Availability Statement

The raw data supporting the conclusions of this article will be made available by the authors upon request.

## Ethics Statement

The protocol, consent form, and study documents were approved by the institutional review boards at the study sites. Participation was voluntary, and written informed consent was obtained from all participants. The patients/participants provided their written informed consent to participate in this study.

## Author Contributions

Conceptualization: AK, ES, MM. Data curation: GA, AK, ES, AC, MF, and MA-P. Investigation: AM, MR, AS, MA, MA-P, and ES. Formal analysis: MA, BD, JL, SC, VR, ES, TS, MC-S, BA, and AK. Funding acquisition: TS, BA, and AK. Methodology: AKS, PV, BD, JL, SC, VR, ES, TS, MC-S, BA, and AK. Project administration: MF, TS, BA, and AK. Resources: TS, BA, and AK. Software: GA. Supervision: AK and ES. Writing—original draft: AC, GA, ES, MM, TS, MC-S, BA, and AK. Writing—review and editing: all authors. All authors have read and agreed to the submitted version of the manuscript.

## Funding

Departamento de Ciência e Tecnologia, Ministério da Saúde, Brazil and the National Institutes of Allergy and Infectious Diseases, USA. The work of AK, BA, ES, GA, AC, MF, JL, SC, VR, BD, TS, and MC-S was supported by grants from NIH (U01AI069923, R01AI120790). BA, AK, and JL are senior scientists from the Conselho Nacional de Desenvolvimento Científico e Tecnológico (CNPq). MM received a scholarship from CNPq. MA received a scholarship from Fundação de Amparo à Pesquisa do Estado da Bahia (FAPESB). MA-P received a research fellowship from the Coordenação de Aperfeiçoamento de Pessoal de Nível Superior (CAPES, finance code: 001). The funders had no role in study design, data collection and analysis, decision to publish, or preparation of the manuscript.

## Conflict of Interest

The authors declare that the research was conducted in the absence of any commercial or financial relationships that could be construed as a potential conflict of interest.
