# Supplementary material for: Pre-Treatment Neutrophil Count as a Predictor of Antituberculosis Therapy Outcomes: A Multicenter Prospective Cohort Study
Source: Front Immunol. 2021 Jul 2;12:661934. doi: 10.3389/fimmu.2021.661934 (PMC8284392; doi:10.3389/fimmu.2021.661934)
Supplement: Supplementary file 1 [file DataSheet_1.docx]

**Pre-treatment absolute neutrophil count as a predictor of antituberculosis therapy outcomes: a multicenter prospective cohort study**

**Table S1. Sociodemographic characteristics, stratified by HIV status.**

|  | **HIV seronegative** | **HIV seropositive** | **P-value** |
| --- | --- | --- | --- |
|  | ***N=764*** | ***N=172*** |  |
| Age at enrollment: | 36.0 [25.0;50.0] | 35.0 [27.0;42.0] | 0.035 |
| Race/skin color: Non-black | 546 (71.6%) | 146 (84.9%) | <0.001 |
| Sex: Male | 486 (63.6%) | 133 (77.3%) | <0.001 |
| Smoking: Yes | 185 (24.2%) | 28 (16.3%) | 0.022 |
| Smoking (years) | 15.5 [6.00;25.2] | 13.0 [4.00;21.2] | 0.015 |
| Alcohol: Yes | 368 (48.2%) | 56 (32.6%) | <0.001 |
| BCG scar: Yes | 674 (88.3%) | 135 (78.5%) | 0.001 |
| Alcohol (years) | 14.0 [6.00;28.0] | 13.0 [7.00;21.0] | 0.091 |
| X-ray cavitation: Yes | 430 (59.6%) | 35 (22.6%) | 0.000 |
| Literate: Yes | 40 (5.24%) | 6 (3.51%) | 0.357 |
| Education (years): | 9.00 [6.00;12.0] | 10.0 [6.00;12.0] | 0.301 |
| Income: more than minimum wage | 247 (33.1%) | 52 (30.6%) | 0.531 |
| HIV treatment: Yes | 0 (0.00%) | 127 (90.1%) |  |
| CD4 (cells/mm^3^): | . [.;.] | 135 [63.5;298] |  |
| Study site: |  |  |  |
| A | 172 (22.5%) | 7 (4.07%) | Ref. |
| B | 137 (17.9%) | 130 (75.6%) | 0.000 |
| C | 238 (31.2%) | 2 (1.16%) | 0.041 |
| D | 95 (12.4%) | 29 (16.9%) | <0.001 |
| E | 122 (16.0%) | 4 (2.33%) | 0.758 |

**Note**: Values are represented as frequency (%) or median with interquartile range (IQR). 95% confidence intervals are displayed. P-values computed via Wald tests. Smoking: current smoker (Yes/No); Alcohol: current (Yes/No) Literate: literacy (Yes/No); Income: monthly salary. CD4: CD4 count at baseline. Study site: sites covered by RePORT. **Abbreviations**. OR: odds ratio. Study sites: A – Caxias Health Center/Rio de Janeiro, B- Tropical Medicine Foundation/Manaus; C: Jose Silveira Foundation/Salvador; D: Evandro Chagas Institute-Rio de Janeiro; E: Rocinha – Municipality of Rio de Janeiro.

**Table S2: Laboratory values, stratified by HIV status.**

|  | **HIV-seronegative** | **HIV-seropositive** | **P-value** |
| --- | --- | --- | --- |
|  | ***N=764*** | ***N=172*** |  |
| Neutrophils (10^3^/mm^3^) | 6.12 [4.63;8.09] | 5.05 [3.59;7.25] | 0.005 |
| Glycosylated Hemoglobin (%) | 5.80 [5.50;6.40] | 5.90 [5.40;6.38] | <0.001 |
| Platelet (10^4^/mm^3^) | 39.0 [31.4;48.1] | 36.2 [28.8;46.8] | 0.066 |
| Lymphocytes (10^2^/mm^3^) | 16.0 [12.5;20.0] | 12.0 [7.69;16.3] | <0.001 |
| Leukocytes (10^3^/mm^3^) | 8.70 [6.87;10.8] | 6.91 [5.63;9.59] | <0.001 |
| Hemoglobin (g/dL) | 12.3 [11.2;13.5] | 10.6 [8.80;12.0] | <0.001 |
| Anemia: Yes | 406 (53.6%) | 128 (74.9%) | <0.001 |
| Glycosylated Hemoglobin (%): |  |  |  |
| < 5.7 | 347 (45.8%) | 69 (40.6%) | Ref. |
| 5.7-6.5 | 232 (30.6%) | 65 (38.2%) | 0.076 |
| ≥ 6.5 | 178 (23.5%) | 36 (21.2%) | 0.934 |

**Note**: Values are represented as frequency (%) or median with interquartile range (IQR). 95% confidence intervals are displayed. P-values computed via Wald tests. Anemia: hemoglobin levels <12 g/dL for female and <13.5 g/dL for male. **Abbreviations**. OR: odds ratio

|  | **Negative sputum smear result at baseline** | **Positive sputum smear result at baseline** | **P-value** |
| --- | --- | --- | --- |
|  | ***N=180*** | ***N=747*** |  |
| Age at enrollment: | 34.0 [24.8;45.2] | 36.0 [25.0;49.0] | 0.139 |
| Race/skin color: Non-black | 138 (76.7%) | 545 (73.1%) | 0.325 |
| Sex: Male | 119 (66.1%) | 494 (66.1%) | 0.991 |
| HIV: Yes | 61 (33.9%) | 110 (14.7%) | <0.001 |
| HIV treatment: Yes | 41 (82.0%) | 85 (85.0%) | 0.637 |
| CD4 (cells/mm^3^) | 110 [51.0;304] | 145 [72.0;282] | 0.822 |
| Smoking: Yes | 30 (16.7%) | 183 (24.5%) | 0.022 |
| Smoking (years) | 14.0 [5.00;25.0] | 15.0 [5.75;25.0] | 0.335 |
| Alcohol: Yes | 67 (37.2%) | 354 (47.4%) | 0.014 |
| BCG scar: Yes | 149 (82.8%) | 653 (87.5%) | 0.101 |
| Alcohol (years) | 13.0 [7.00;25.5] | 13.0 [6.00;27.0] | 0.959 |
| X-ray cavitation: Yes | 54 (31.2%) | 406 (58.5%) | <0.001 |
| Literate: Yes | 6 (3.33%) | 39 (5.23%) | 0.295 |
| Education (years): | 10.0 [7.00;12.0] | 9.00 [6.00;12.0] | 0.074 |
| Income: More than minimum wage | 61 (34.9%) | 236 (32.2%) | 0.500 |
| Study site: |  |  |  |
| A | 28 (15.6%) | 151 (20.2%) | Ref. |
| B | 66 (36.7%) | 201 (26.9%) | 0.021 |
| C | 26 (14.4%) | 214 (28.6%) | 0.152 |
| D | 38 (21.1%) | 86 (11.5%) | 0.002 |
| E | 22 (12.2%) | 95 (12.7%) | 0.481 |

**Table S3: Sociodemographic characteristics and laboratory values, stratified by sputum smear result at baseline**

**Note**: Values are represented as frequency (%) or median with interquartile range (IQR). 95% confidence intervals are displayed. P-values computed via Wald tests. Smoking: current smoker (Yes/No); Alcohol: current (Yes/No) Literate: literacy (Yes/No); Income: monthly salary. CD4: CD4 count at baseline. Study site: sites covered by RePORT.. **Abbreviations**. OR: odds ratio. Study sites: A – Caxias Health Center/Rio de Janeiro, B- Tropical Medicine Foundation/Manaus; C: Jose Silveira Foundation/Salvador; D: Evandro Chagas Institute-Rio de Janeiro; E: Rocinha – Municipality of Rio de Janeiro

**Table S4: Laboratory values, stratified by sputum smear result at baseline**

|  | **Negative sputum smear result at baseline** | **Positive sputum smear result at baseline** | **P-value** |
| --- | --- | --- | --- |
|  | ***N=180*** | ***N=747*** |  |
| Neutrophils (10^3^/mm^3^) | 5.22 [3.75;6.96] | 6.17 [4.56;8.16] | 0.001 |
| Glycosylated Hemoglobin (%) | 5.80 [5.40;6.20] | 5.90 [5.50;6.40] | 0.050 |
| Platelet (10^4^/mm^3^) | 36.2 [27.5;45.5] | 39.0 [31.5;48.7] | 0.003 |
| Lymphocytes (10^2^/mm^3^) | 14.8 [10.7;19.1] | 15.6 [11.9;19.5] | 0.338 |
| Leukocytes (10^3^/mm^3^) | 7.47 [5.90;9.83] | 8.63 [6.80;10.8] | <0.001 |
| Hemoglobin (g/dL) | 12.1 [10.2;13.6] | 12.1 [10.8;13.3] | 0.485 |
| Anemia: Yes | 104 (57.8%) | 430 (57.6%) | 0.961 |
| Glycosylated Hemoglobin (%): |  |  |  |
| < 5.7 | 88 (48.9%) | 327 (43.9%) | Ref. |
| 5.7-6.5 | 58 (32.2%) | 238 (31.9%) | 0.604 |
| ≥ 6.5 | 34 (18.9%) | 180 (24.2%) | 0.109 |

**Note**: Values are represented as frequency (%) or median with interquartile range (IQR). 95% confidence intervals are displayed. P-values computed via Wald tests. Anemia: hemoglobin levels <12 g/dL for female and <13.5 g/dL for male. **Abbreviations**. OR: odds ratio

**Table S5: Sociodemographic characteristics, stratified by sputum smear result at month 2.**

|  | **Negative sputum smear result at month 2** | **Positive sputum smear result at month 2** | **P - value** |
| --- | --- | --- | --- |
|  | ***N=587*** | ***N=126*** |  |
| Age at enrollment: | 35.0 [25.0;47.5] | 39.5 [31.0;55.0] | <0.001 |
| Race:/skin color: Non-black | 426 (72.7%) | 90 (71.4%) | 0.767 |
| Sex: Male | 384 (65.4%) | 91 (72.2%) | 0.141 |
| HIV: Yes | 110 (18.7%) | 36 (28.6%) | 0.016 |
| HIV treatment: Yes | 88 (90.7%) | 28 (77.8%) | 0.064 |
| CD4 (cells/mm^3^) | 142 [61.5;240] | 91.0 [66.0;380] | 0.867 |
| Smoking: Yes | 120 (20.4%) | 31 (24.6%) | 0.303 |
| Smoking (years) | 15.0 [5.00;25.0] | 18.5 [5.75;30.5] | 0.032 |
| Alcohol: Yes | 268 (45.7%) | 54 (42.9%) | 0.570 |
| BCG scar: Yes | 507 (86.4%) | 104 (82.5%) | 0.271 |
| Alcohol (years) | 12.0 [6.00;25.5] | 19.0 [10.0;36.5] | 0.002 |
| X-ray cavitation: Yes | 271 (50.2%) | 69 (58.5%) | 0.104 |
| Literate: Yes | 26 (4.44%) | 8 (6.35%) | 0.368 |
| Education (years): | 10.0 [6.00;12.0] | 8.00 [5.00;12.0] | 0.087 |
| Income: More than minimum wage | 189 (33.0%) | 38 (30.4%) | 0.574 |
| Study site: |  |  |  |
| A | 60 (10.2%) | 15 (11.9%) | Ref. |
| B | 177 (30.2%) | 57 (45.2%) | 0.446 |
| C | 193 (32.9%) | 37 (29.4%) | 0.437 |
| D | 106 (18.1%) | 14 (11.1%) | 0.122 |
| E | 51 (8.69%) | 3 (2.38%) | 0.019 |

**Note**: Values are represented as frequency (%) or median with interquartile range (IQR). 95% confidence intervals are displayed. P-values computed via Wald tests. Smoking: current smoker (Yes/No); Alcohol: current (Yes/No) Literate: literacy (Yes/No); Income: monthly salary. CD4: CD4 count at baseline. Study site: sites covered by RePORT. **Abbreviations**. OR: odds ratio. Study sites: A – Caxias Health Center/Rio de Janeiro, B- Tropical Medicine Foundation/Manaus; C: Jose Silveira Foundation/Salvador; D: Evandro Chagas Institute-Rio de Janeiro; E: Rocinha – Municipality of Rio de Janeiro

**Table S6: Baseline laboratory values, stratified by sputum smear result at month 2.**

|  | **Negative sputum smear result at month 2** | **Positive sputum smear result at month 2** | **P - value** |
| --- | --- | --- | --- |
|  | ***N=587*** | ***N=126*** |  |
| Neutrophils (10^3^/mm^3^) | 6.01 [4.49;8.02] | 6.38 [4.27;7.92] | 0.926 |
| Glycosylated Hemoglobin (%) | 5.80 [5.50;6.30] | 6.00 [5.60;6.70] | 0.128 |
| Platelet (10^4^/mm^3^) | 38.5 [30.5;47.8] | 39.0 [32.5;49.1] | 0.245 |
| Lymphocytes (10^2^/mm^3^) | 15.5 [11.7;19.3] | 13.7 [11.0;19.0] | 0.055 |
| Leukocytes (10^3^/mm^3^) | 8.42 [6.60;10.5] | 8.70 [6.31;10.7] | 0.967 |
| Hemoglobin (g/dL) | 12.2 [10.8;13.4] | 11.5 [10.2;12.8] | 0.343 |
| Anemia: Yes | 328 (56.3%) | 86 (68.3%) | 0.013 |
| Glycosylated Hemoglobin (%): |  |  |  |
| < 5.7 | 263 (45.3%) | 42 (33.3%) | Ref. |
| 5.7-6.5 | 190 (32.7%) | 47 (37.3%) | 0.061 |
| ≥ 6.5 | 128 (22.0%) | 37 (29.4%) | 0.019 |

**Note**: Values are represented as frequency (%) or median with interquartile range (IQR). 95% confidence intervals are displayed. P-values computed via Wald tests. Anemia: hemoglobin levels <12 g/dL for female and <13.5 g/dL for male. **Abbreviations**. OR: odds ratio

**Table S7: Sociodemographic characteristics, stratified by MTB culture result at month 2.**

|  | **Negative culture**  **at month 2** | **Positive culture**  **at month 2** | **P-value** |
| --- | --- | --- | --- |
|  | ***N=642*** | ***N=40*** |  |
| Age at enrollment: | 36.0 [25.0;48.0] | 50.5 [34.8;58.5] | <0.001 |
| Race/skin color: Non-black | 462 (72.1%) | 29 (72.5%) | 0.973 |
| Sex: Male | 424 (66.0%) | 30 (75.0%) | 0.248 |
| HIV: Yes | 132 (20.6%) | 7 (17.5%) | 0.668 |
| HIV treatment: Yes | 107 (87.7%) | 6 (100%) | . |
| Smoking: Yes | 136 (21.2%) | 9 (22.5%) | 0.823 |
| Smoking (years) | 15.0 [5.00;25.0] | 30.0 [15.0;38.0] | 0.006 |
| Alcohol: Yes | 294 (45.8%) | 16 (40.0%) | 0.483 |
| BCG scar: Yes | 555 (86.4%) | 32 (80.0%) | 0.268 |
| Alcohol (years) | 13.0 [6.00;27.0] | 22.5 [11.2;33.5] | 0.183 |
| X-ray cavitation: Yes | 314 (52.7%) | 19 (54.3%) | 0.858 |
| Literate: Yes | 32 (4.99%) | 3 (7.50%) | 0.481 |
| Education (years): | 10.0 [6.00;12.0] | 8.00 [6.00;11.0] | 0.475 |
| Income: More than minimum wage | 208 (33.2%) | 10 (25.0%) | 0.291 |
| Study site: |  |  |  |
| A | 60 (9.35%) | 9 (22.5%) | Ref. |
| B | 201 (31.3%) | 14 (35.0%) | 0.102 |
| C | 218 (34.0%) | 9 (22.5%) | 0.012 |
| D | 111 (17.3%) | 6 (15.0%) | 0.068 |
| E | 52 (8.10%) | 2 (5.00%) | 0.079 |

**Note**: Values are represented as frequency (%) or median with interquartile range (IQR). 95% confidence intervals are displayed. P-values computed via Wald tests. Smoking: current smoker (Yes/No); Alcohol: current (Yes/No) Literate: literacy (Yes/No); Income: monthly salary. CD4: CD4 count at baseline. Study site: sites covered by RePORT. **Abbreviations**. OR: odds ratio. Study sites: A – Caxias Health Center/Rio de Janeiro, B- Tropical Medicine Foundation/Manaus; C: Jose Silveira Foundation/Salvador; D: Evandro Chagas Institute-Rio de Janeiro; E: Rocinha – Municipality of Rio de Janeiro

**Table S8: Baseline Laboratory values, stratified by MTB culture result at month 2.**

|  | **Negative culture at month 2** | **Positive culture at month 2** | **OR** | **P-value** |
| --- | --- | --- | --- | --- |
|  | ***N=642*** | ***N=40*** |  |  |
| Neutrophils (10^3^/mm^3^) | 6.02 [4.44;8.00] | 6.63 [4.94;8.77] | 1.05 [0.95;1.15] | 0.356 |
| Glycosylated Hemoglobin (%) (%) | 5.90 [5.50;6.40] | 5.90 [5.57;6.38] | 1.02 [0.88;1.19] | 0.778 |
| Platelet (10^4^/mm^3^) | 38.7 [30.8;47.9] | 41.7 [36.7;54.1] | 1.02 [1.00;1.04] | 0.046 |
| Lymphocytes (10^2^) | 15.5 [11.4;19.4] | 12.6 [11.2;16.1] | 0.95 [0.90;1.00] | 0.056 |
| Leukocytes (10^3^/mm^3^) | 8.48 [6.53;10.5] | 8.79 [7.22;12.3] | 1.04 [0.96;1.13] | 0.366 |
| Hemoglobin (g/dL) | 12.1 [10.7;13.4] | 11.6 [10.5;12.2] | 0.88 [0.75;1.03] | 0.113 |
| Anemia: Yes | 369 (57.8%) | 28 (70.0%) | 1.69 [0.86;3.52] | 0.131 |
| Glycosylated Hemoglobin (%) |  |  |  |  |
| < 5.7 | 272 (42.8%) | 17 (42.5%) | Ref. | Ref. |
| 5.7-6.5 | 218 (34.3%) | 13 (32.5%) | 0.96 [0.44;2.02] | 0.908 |
| ≥ 6.5 | 146 (23.0%) | 10 (25.0%) | 1.10 [0.47;2.45] | 0.815 |

**Note**: Values are represented as frequency (%) or median with interquartile range (IQR). 95% confidence intervals are displayed. P-values computed via Wald tests. Anemia: hemoglobin levels <12 g/dL for female and <13.5 g/dL for male. **Abbreviations**. OR: odds ratio

**Table S9: Association between neutrophil count at baseline and the outcome of interest.**

| **Outcome variable** | **Population** | **Sample size** | **Multivariable analysis**† |
| --- | --- | --- | --- |
| Unfavorable TB treatment outcome  (excluding non-TB deaths) | HIV-seronegative | 570 | 1.13 [1.01; 1.28] |
|  | HIV-seropositive | 103 | 1.01 [0.77; 1.33] |
| Unfavorable TB treatment outcome  (adjusting for leukocytes) | HIV-seronegative | 577 | 1.21 [1.09; 1.34] |
|  | HIV-seropositive | 114 | 1.01 [0.81; 1.27] |
| Unfavorable TB treatment outcome  (9 months of follow-up) | HIV-seronegative | 677 | 1.15 [1.03; 1.29] |
|  | HIV-seropositive | 135 | 0.92 [0.70; 1.21] |
| Unfavorable TB treatment outcome  (Mixed-effects model) | HIV-seronegative | 577 | 1.16 [1.04; 1.29] |
|  | HIV-seropositive | 114 | 0.98 [0.77; 1.24] |

**Note**: † Model adjusting for the propensity score, which was regressed on the following covariates: sex, age, race, smoking status, alcohol consumption, education level, income status, cavitation on chest radiograph, study site, HIV status (only when all patients were used), DOT, and (log-transformed) platelets, lymphocytes, glycated hemoglobin and hemoglobin values (plus log-transformed leukocytes, when indicated); results expressed in odds ratios (OR) with 95% confidence intervals. Study sites were used as random effects for the mixed-effects analysis.


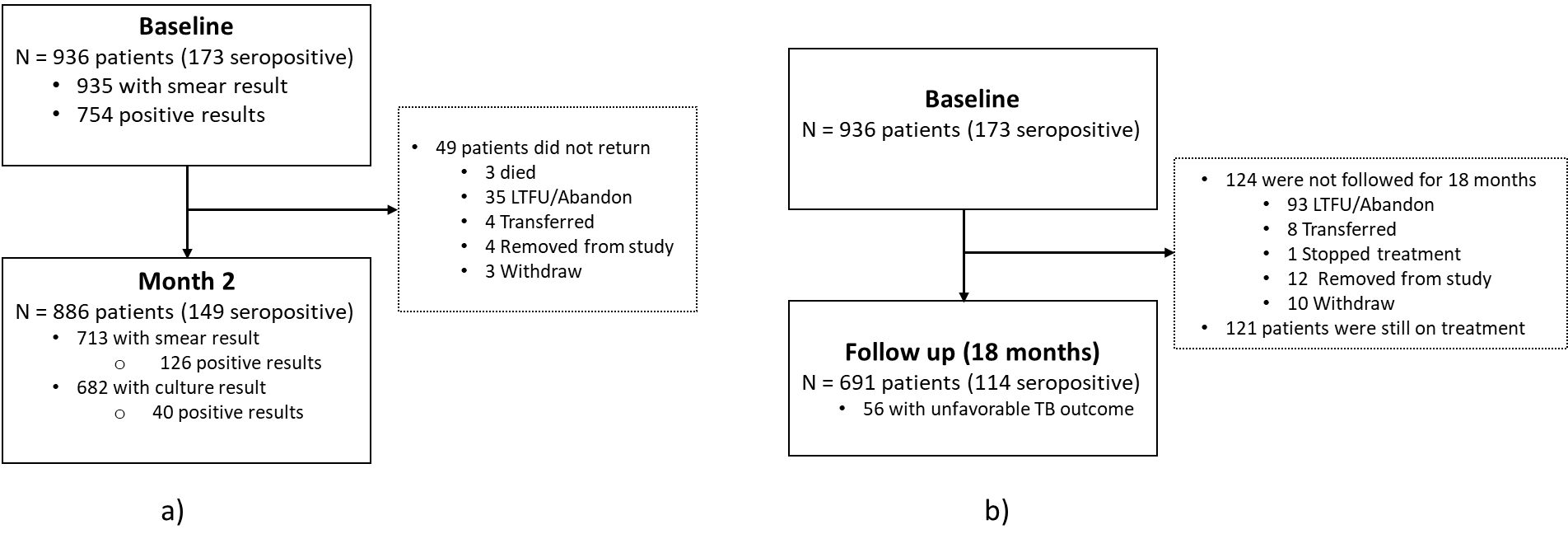
 **Figure S1: Sample size used for all four analysis. A)** shows the sample size for assessing the association between neutrophil and smear at baseline, smear result at month 2, and culture result at month 2; **B)** displays the sample size for to assess the association between baseline neutrophil and TB treatment**.**

**
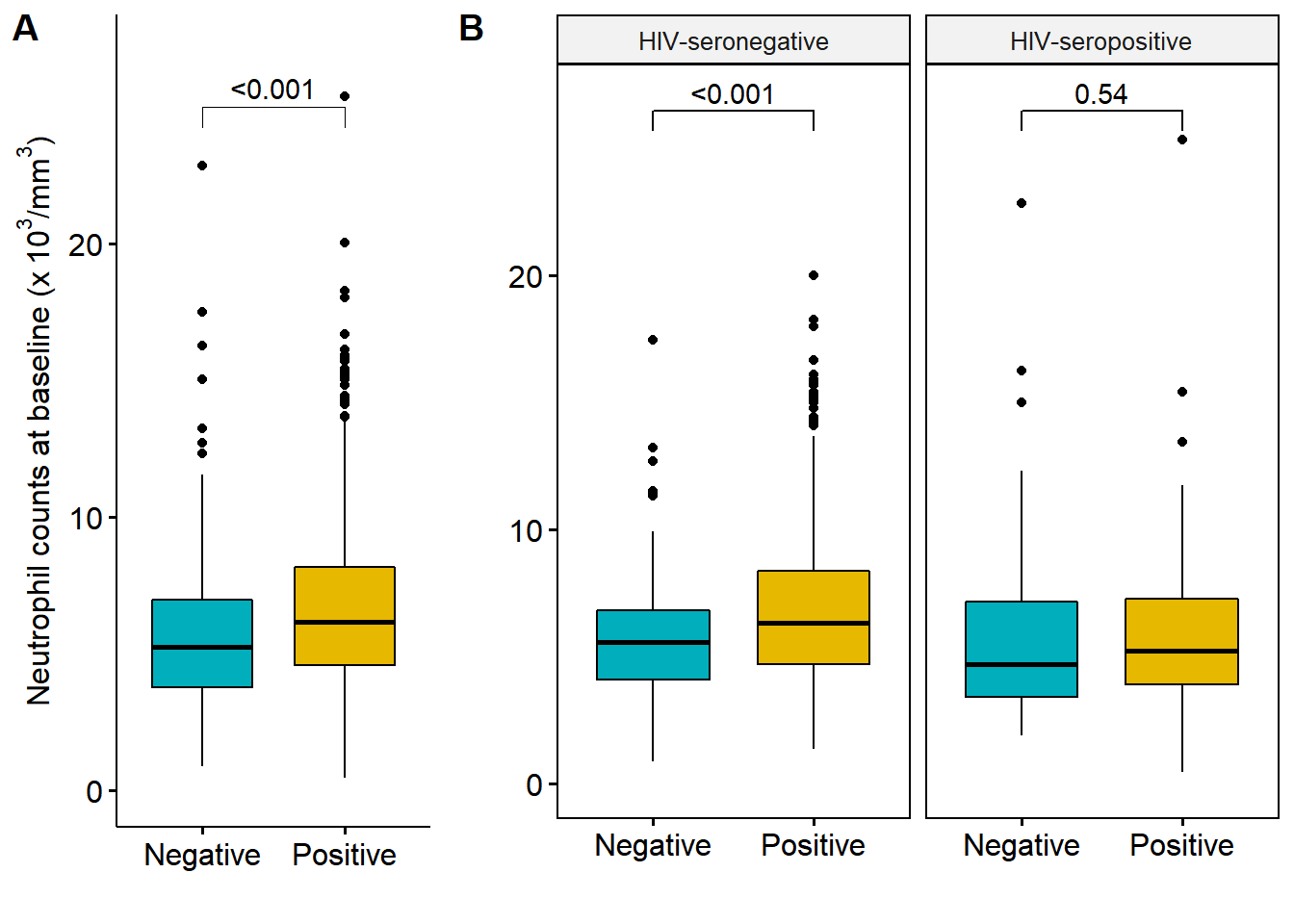
**

**Figure S2: Neutrophil count at baseline by sputum smear result. (A)** Comparison of neutrophil count at baseline by sputum smear result at baseline, among 927 patients (747 with positive smear); **(B)** comparisons of neutrophil count by sputum smear result, both at baseline and at month 2, stratified by HIV status (756 seronegative patients, 637 with positive smear; 171 seropositive patients, 110 with positive smear). P-value computed via Wald test.

**
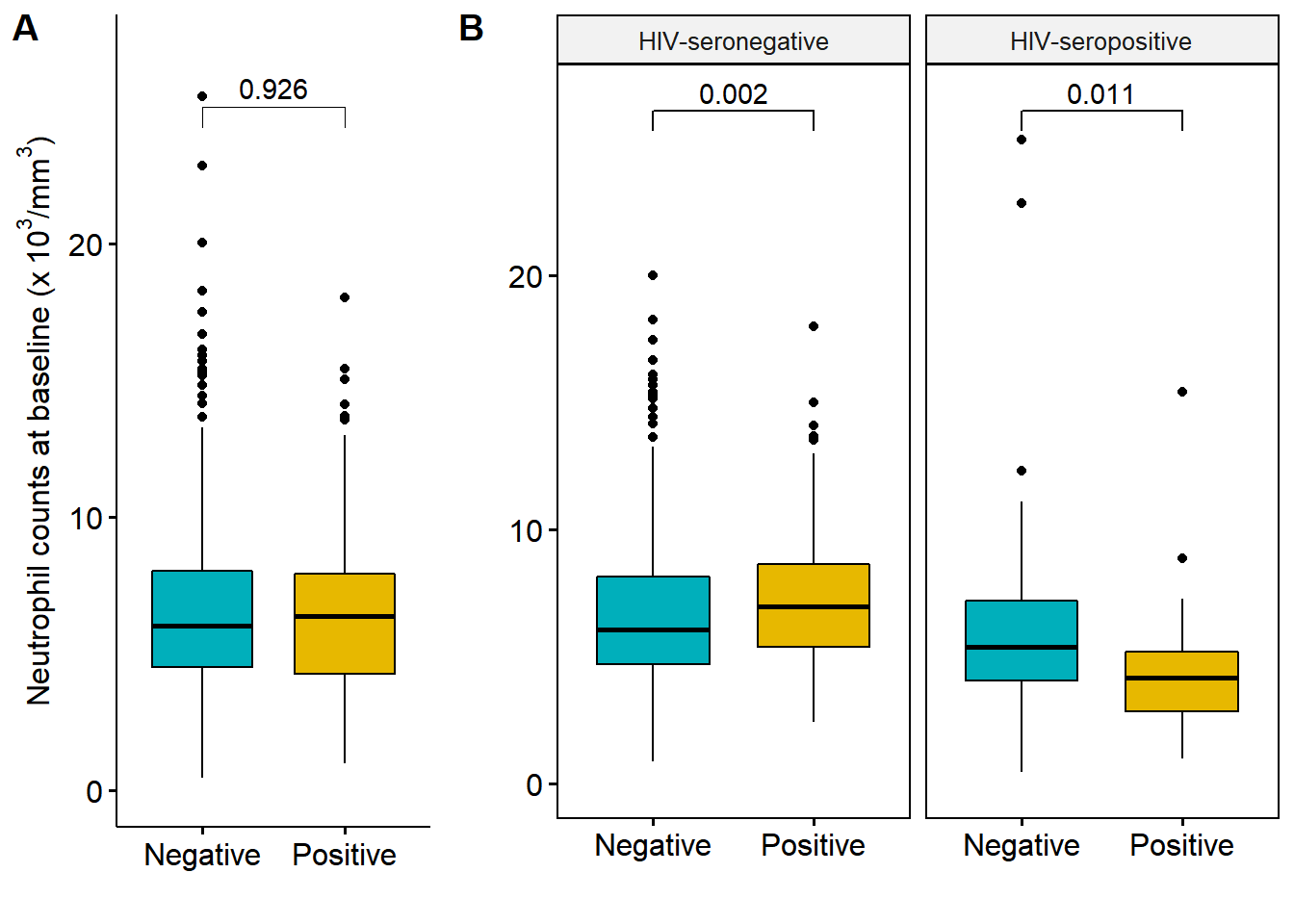
**

**Figure S3: Neutrophil count at baseline by sputum smear result at month 2.**

**(A)** Comparison of neutrophil count at baseline by sputum smear result at month 2 for 709 patients (126 with positive smear) and **(B)** stratified by HIV status (564 seronegative patients, 90 with positive smear; 145 seropositive patients, 36 with positive smear). P-value computed via Wald test.

**
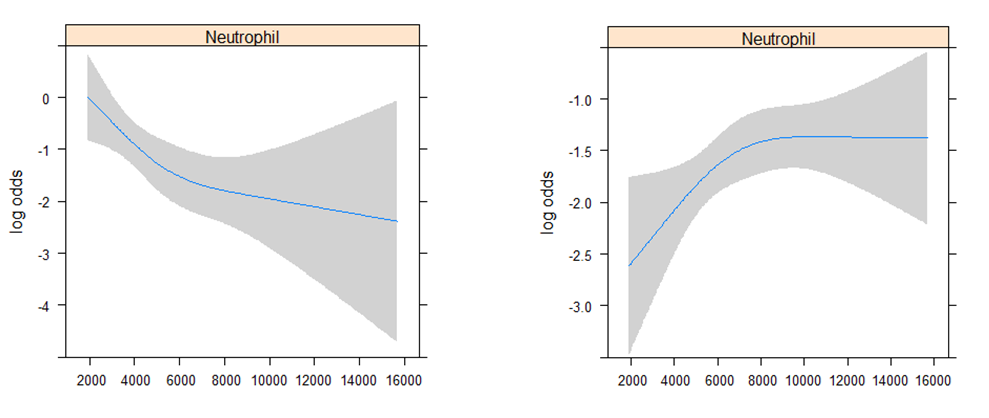
**

**A) B)**

**Figure S4: Log-odds of being smear positive at month 2 by neutrophil counts at month 2. A)** HIV-seropositive and **B)** HIV-seronegative patients. Restricted cubic splines with three knots, equally spaced, were used to alleviate linearity assumptions.

***
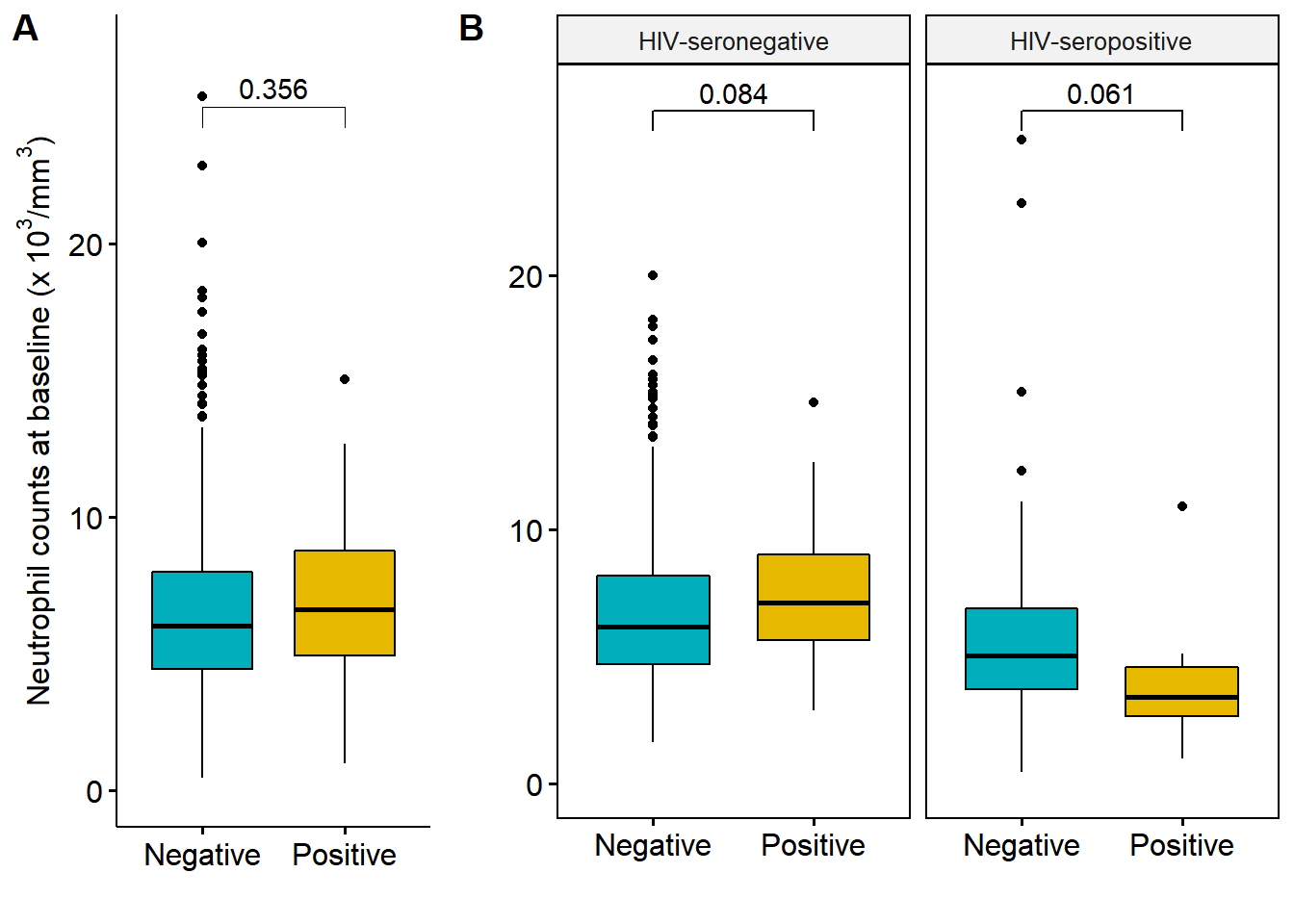
***

**Figure S5: Neutrophil count at baseline by MTB culture result.**

(A) Comparison of neutrophil count at baseline by MTB culture result (positive/negative) at month 2, for 682 patients (40 with positive culture); (B) comparisons of neutrophil count by MTB culture result, stratified by HIV status (540 seronegative patients, 33 with positive smear; 131 seropositive patients, 7 with positive smear). P-value computed via Wald test.
